# Cryptococcus albidus (Naganishia albida) meningitis in a young patient with T-cell acute lymphoblastic leukaemia

**DOI:** 10.1099/acmi.0.000920.v4

**Published:** 2025-10-15

**Authors:** Amber Prasad, Minakshi Singh, Priyal Anand

**Affiliations:** 1 Department of Microbiology, AIIMS Rishikesh, Rishikesh, India

**Keywords:** Cryptococcal meningitis, *Cryptococcus albidus*, Encephalitis, *Naganishia albida*, T-cell acute lymphoblastic leukaemia (T-ALL)

## Abstract

*Cryptococcus albidus*, an emerging pathogen, poses diagnostic and therapeutic challenges, especially in immunocompromised patients. We report a case of *C. albidus* meningitis in a young T-cell acute lymphoblastic leukaemia patient, initially suspected to have Herpes Simplex Virus (HSV) encephalitis. CSF analysis confirmed *C. albidus*, leading to antifungal therapy with liposomal amphotericin B and flucytosine, resulting in clinical improvement. Elevated procalcitonin levels suggest a potential role in fungal infections. This case underscores the importance of early identification and appropriate treatment in *C. albidus* meningitis.

## Data Summary

No data were generated during this research, nor are they required for the work to be reproduced.

## Introduction

*Cryptococcus* species are yeasts belonging to the basidiomycetous fungi, recognized as causative agents for various diseases. Among these, *Cryptococcus neoformans* and *Cryptococcus gattii* are widely acknowledged as the most prevalent pathogenic species [1,3]. Nonetheless, there has been a recent upsurge in infections attributed to non-neoformans cryptococcal species like *Papiliotrema laurentii* (previously known as *Cryptococcus laurentii*) and *Naganishia albida* (synonym *Cryptococcus albidus*) [4,6]. The primary sources of *C. albidus* are considered to be environmental, particularly residues found in the canopies of specific trees [7]. *C. albidus* is an uncommon cause of fungal meningitis, especially in immunocompromised patients, such as those with haematological malignancies. T-cell acute lymphoblastic leukaemia (T-ALL) is associated with immunosuppression, predisposing patients to opportunistic infections [8]. This case highlights the importance of considering rare pathogens in the differential diagnosis of meningitis, particularly in high-risk populations.

## Case presentation

A 16-year-old male with a history of T-ALL presented with a 4-day history of headache, followed by 3 days of fever, vomiting and decreased appetite. The fever ranged from 100 to 102 °F, relieved by antipyretics, and vomiting was non-bilious, non-bloody, with food particles and relieved by antiemetics. The headache was frontal, moderate to severe and not fully relieved by medication. He also had three to four episodes of loose stools 2 days prior but no abdominal pain, cough, rash, bleeding or haematuria.

On examination, he was oriented, with normal tone and power in both upper and lower limbs. Initial tests included complete blood count, liver function test, kidney function test, procalcitonin and blood and urine cultures. Persistent symptoms led to an Magnetic Resonance Imaging (MRI) and Computed Tomography (CT) Scan of the brain. The contrast-enhanced MRI with MR venography revealed T2/Flair hyperintensities with diffuse restriction and post-contrast enhancement in the brain parenchyma, suggesting the likelihood of HSV encephalitis, though the CT scan showed no significant abnormalities, given its lower sensitivity for Cerebrospinal Fluid (CNS) infections. Cerebrospinal Fluid (CSF) analysis included microscopy, India ink staining, bacterial and fungal cultures, ZN stain and cartridge-based nucleic-acid amplification test (Cepheid GeneXpert) for tuberculosis. The patient was started on acyclovir for suspected HSV encephalitis and showed initial improvement, though the headache persisted.

**Fig. 1.**
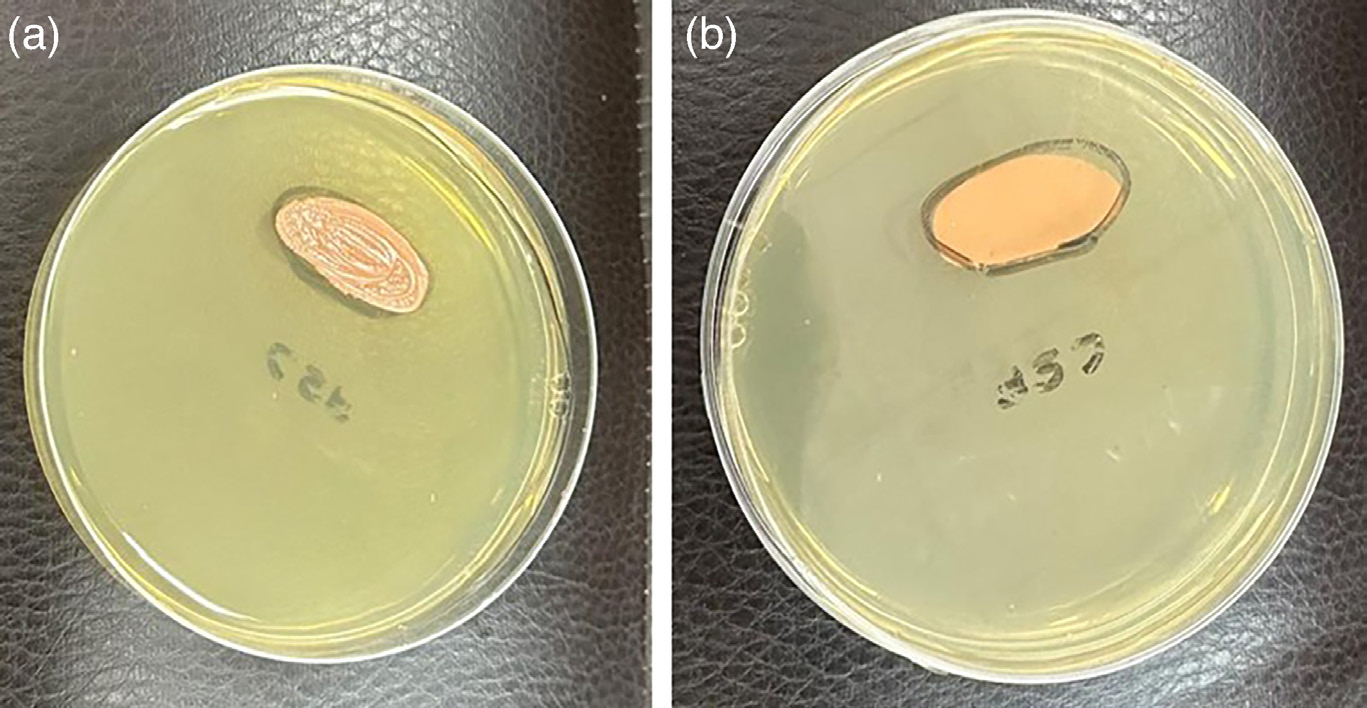
Growth on SDA plate after 4 days of aerobic incubation at 37 °C (Reverse and obverse).

Subsequently, a Biofire Filmarray Meningitis/Encephalitis panel (ME panel; Biomerieux) was done for CSF, targeting six bacteria, seven viruses and one yeast (*C. neoformans*). However, the results indicated the absence of pathogens included in the panel, including HSV 1 and HSV 2. Bacterial culture of CSF revealed no growth after 48 h of aerobic incubation at 37 °C. Fungal culture revealed pale pink-coloured, smooth and mucoid colonies on Sabouraud dextrose agar (SDA) after 4 days of aerobic incubation at 37 and 25 °C (Figs1^3^), subsequently identified as *C. albidus* by VITEK 2 (BioMérieux) using a YST ID card. Antifungal susceptibility testing using the VITEK 2 system (YS08 card), which employs the broth microdilution principle according to Clinical and Laboratory Standards Institute (CLSI) standards for Antimicrobial Susceptibility Testing (AST), confirmed susceptibility to amphotericin B (MIC ≤1 µg ml^−1^) and flucytosine (MIC ≤1 µg ml^−1^). Ophthalmology referral was done in view of ophthalmic complications, but the examination revealed no positive findings. Procalcitonin decreased from 65 to 10.5 ng ml^−1^ after 4 days of treatment, with further normalization to 0.32 ng ml^−1^ on day 10. The patient showed clinical improvement, with resolution of vomiting and headache, though long-term follow-up is needed to monitor for relapse or complications.

**Fig. 2.**
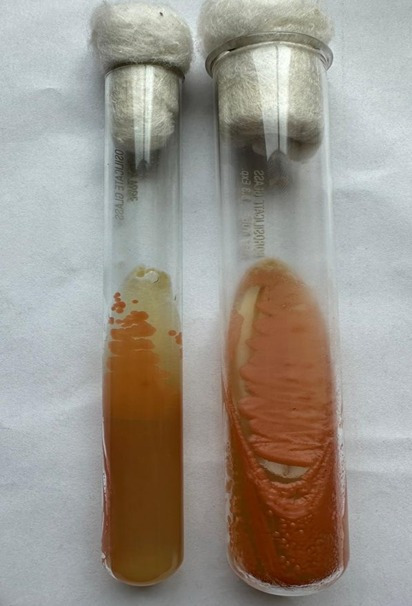
Light pink-coloured smooth, mucoid colonies on SDA tubes at 25 °C (reverse and obverse).

**Fig. 3.**
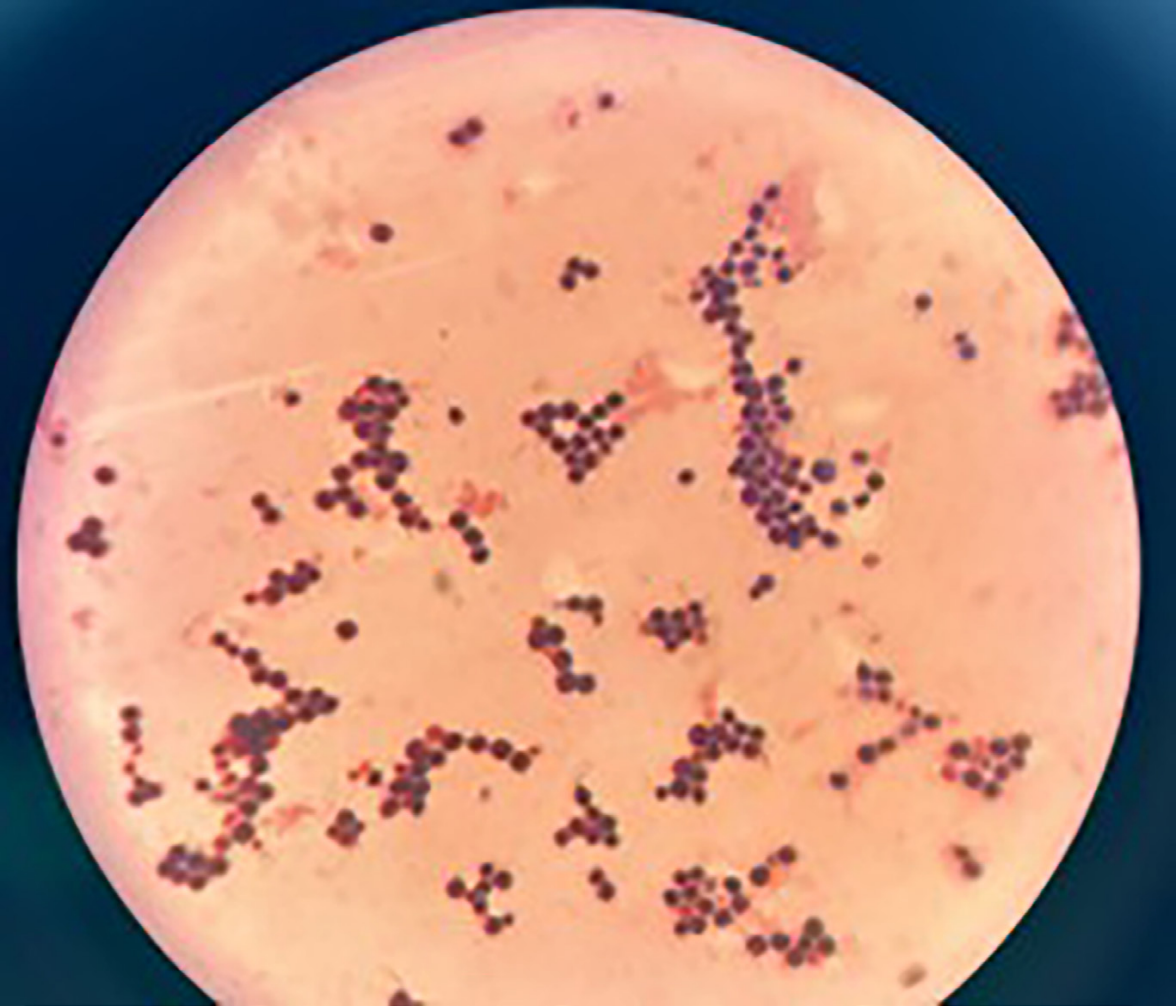
Gram stain from growth on SDA showing rounded yeast cells (100X).

### Diagnostic assessments

Standard protocols for CSF processing were followed according to CLSI guidelines. All CSF samples were processed within 2 h of collection to ensure optimal recovery of pathogens and accurate cell counts. Blood and CSF samples were collected before initiation of any antimicrobial therapy.

CSF opening pressure was 23 cm H2O initially (slightly elevated), which normalized to 18 cm H2O on repeat lumbar puncture performed on day 13 of antifungal therapy (Table 1).

**Table 1.** Cerebrospinal fluid analysis

Parameter	Day 1 (Initial)	Day 13 (Repeat LP)	Reference range
Opening pressure (cm H2O)	23	18	10–20
Appearance	Slightly turbid	Clear	Clear
Cell count (cells μl^−1^)	180	25	<5
Lymphocytes (%)	85	90	>80
Neutrophils (%)	15	10	<1
Protein (mg dl^−1^)	95	60	15–45
Glucose (mg dl^−1^)	35	50	50–75
CSF:serum glucose ratio	0.42	0.65	>0.6
India ink stain	Negative	Negative	–
Cryptococcal antigen*	Negative*	Negative*	–
Gram stain	No organisms/pus cells	No organisms/pus cells	–
Bacterial culture (48 h)	No growth	No growth	–
Fungal culture (SDA)	No growth on days 1–3	*C. albidus* growth on day 4	–
Biofire ME panel	Negative	Not repeated	–

*Note: cryptococcal antigen testing typically targets *C. neoformans* and may be negative in *C. albidus* infections.

Microbiological assessment of CSF: the CSF specimen was centrifuged at 1,000 ***g*** for 5–10 min, and the supernatant was used for cryptococcal antigen testing. For direct microscopy, one drop of the sediment was placed to prepare an India ink mount. The remaining sediment was transferred into a tube containing the remaining CSF and mixed. Several drops of the sediment were transferred to a slide to make a thick smear. The smear was air-dried, heat-fixed and then Gram staining was performed. No pus cells and no micro-organisms were seen in the direct Gram stain of the CSF deposit, likely due to the lower sensitivity of Gram stain compared to culture. The smear was examined microscopically using the 40X and 100X objectives. A KOH mount was prepared using the resuspended sediment sample and observed under 10X and 40X objectives.

For bacterial culture, the resuspended sediment was inoculated onto blood agar, MacConkey agar and chocolate agar using the streak culture method. Sample was also inoculated in Brain Heart Infusion (BHI) broth and incubated aerobically at 37 °C. The chocolate agar plates were incubated in a candle jar at 37 °C, while the blood agar plates were incubated aerobically at 37 °C.

*Candida* spp. and *C. neoformans* are the most common causes of fungal (yeast) meningitis. These were initially ruled out, as no growth was observed on blood agar and chocolate agar, where they typically grow. Further confirmation was obtained through the Biofire ME panel, which specifically targets *C. neoformans* and is considered a gold standard for rapid pathogen detection. However, this panel does not include *C. albidus*, explaining why initial screening was negative despite the presence of this rare cryptococcal species.

For fungal culture, the resuspended sediment was inoculated onto SDA plates and tubes using both central inoculation on plates and the streak method in tubes, with duplicate cultures incubated at 25 and 37 °C. The central inoculation on plates allowed observation of overall colony morphology and pigmentation, while the streak inoculation in tubes (as shown in Fig. 2) provided well-isolated single colonies necessary for accurate yeast identification.

Although the Biofire ME panel has limitations, including potential false negatives and lower sensitivity compared to traditional methods for some pathogens, it represents the best available rapid diagnostic tool in our resource-limited setting. While we recognize that bacterial culture is not a definitive gold standard and cannot entirely exclude all bacterial infections, we ruled out the common causes of bacterial meningitis through comprehensive culture methods and the Biofire ME panel, which includes the more common bacterial pathogens (*Escherichia coli* K1, *Haemophilus influenzae*, *Listeria monocytogenes*, *Neisseria meningitidis*, *Streptococcus agalactiae* and *Streptococcus pneumoniae*).

**Fig. 4.**
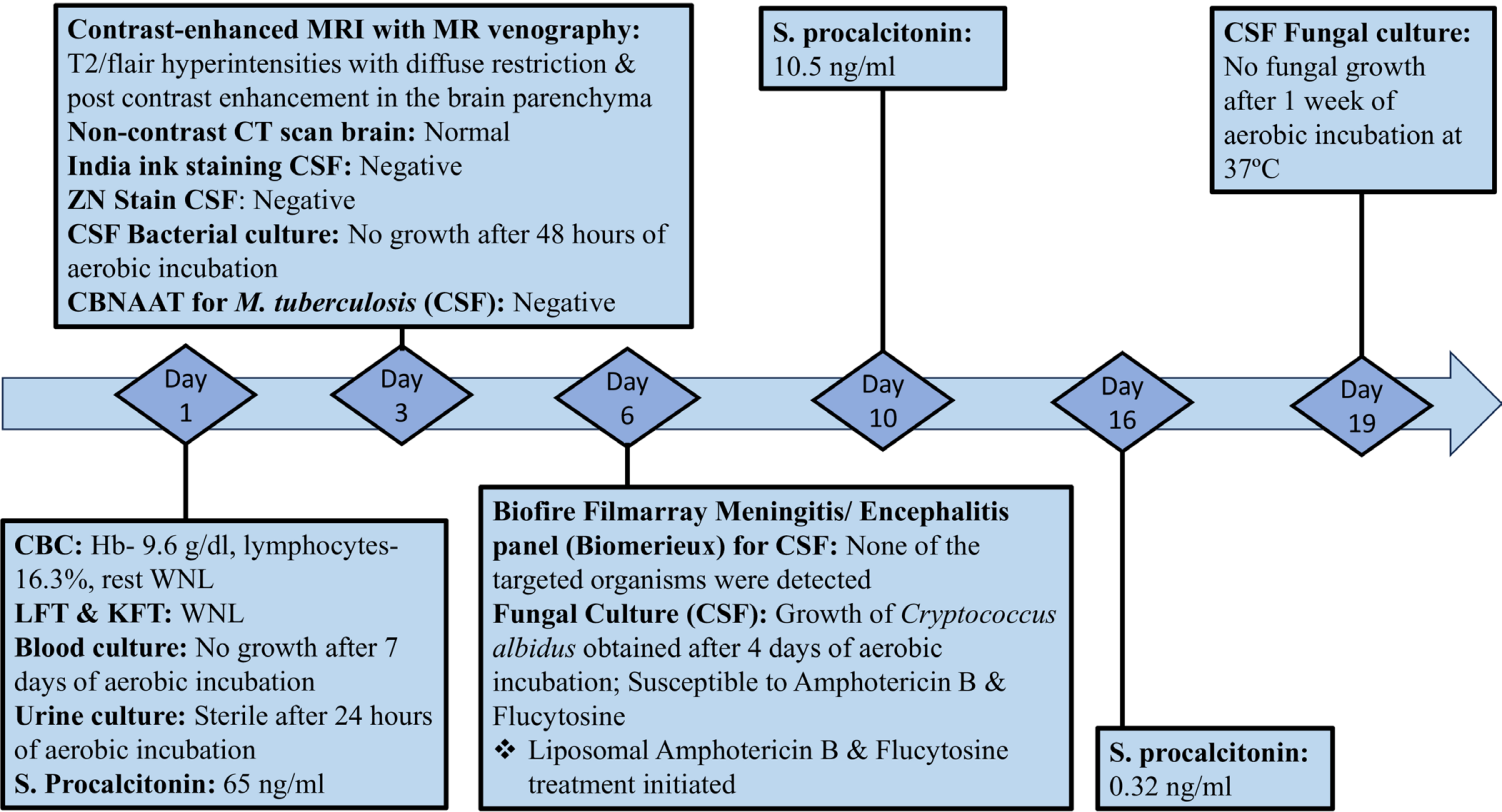
Diagnostic timeline. Day refers to day of admission and day of investigation sent to laboratory. CBC, complete blood count; LFT, liver function test; KFT, kidney function test; CBNAAT, cartridge-based nucleic-acid amplification test.

The diagnostic assessments are shown in Fig. 4.

### Differential diagnoses

1. Viral meningitis was ruled out by the Biofire Filmarray ME panel (Biomerieux).
2. *C. neoformans* was ruled out by the Biofire Filmarray ME panel (Biomerieux).
3. Bacterial meningitis was ruled out, as there was no bacterial growth on blood agar and chocolate agar after 48 h of aerobic incubation at 37 °C.

### Therapeutic interventions

Following identification of *C. albidus*, treatment was initiated with induction therapy consisting of intravenous liposomal amphotericin B and flucytosine for 15 days. The patient received a 50 mg injection of liposomal amphotericin B diluted in 250 ml of NS over 4 h daily, supplemented with 10 mmol of potassium chloride and 4 mmol of magnesium sulphate. Additionally, tablet flucytosine was initiated, to be taken four times daily in divided doses, amounting to a total of 5,250 mg per day. This was continued for 14 days.

### Follow-up and outcome

The patient had significant symptomatic improvement – as the complaints of headache and vomiting subsided and intracranial pressure was normalized. After completion of induction therapy and negative CSF fungal culture, consolidation therapy was started with fluconazole, 800 mg PO daily. The patient was advised for follow-up and for regular monitoring of liver enzymes at least weekly.

## Discussion

This case underscores the challenges in diagnosing and managing *C. albidus* meningitis, particularly in immunocompromised patients. Prompt identification and appropriate antifungal therapy are essential for favourable outcomes. The immune response plays a critical role in *Cryptococcus* infection pathogenesis, as highlighted by studies *on C. neoformans*. Adult meningitis studies in high-prevalence settings provide valuable insights into the clinical spectrum and outcomes of cryptococcal disease. This case report contributes to the existing literature by highlighting *C. albidus* as an emerging pathogen in meningitis cases, especially among immunocompromised individuals. While procalcitonin is commonly utilized as an indicator of bacterial infection, our case presented a notable deviation from this norm. Specifically, we observed a significant elevation in procalcitonin levels associated with fungal meningitis. Remarkably, these levels exhibited a marked decrease following the initiation of antifungal therapy. Elevated procalcitonin levels have been observed in patients with invasive fungal infections, particularly those caused by *Candida species* [9]. However, the significance of procalcitonin levels in cryptococcal infections remains largely unexplored.

Cunha and Lusins reported a case of *C. albidus* meningitis in an adult male patient in 1973. CSF India ink was positive, and culture revealed growth of *C. albidus*. The patient was started on liposomal amphotericin B and symptomatically improved after 1 week of treatment [10]. A case of cryptococcal albidus meningitis in an immunocompromised adult male patient who was already receiving corticosteroids was reported by J.C. Melo *et al.* from the USA in 1980 [11]. CSF was negative for India ink and the cryptococcal antigen test. Treatment with amphotericin B was started, but unfortunately, the patient had cardiorespiratory arrest on the 89th day of his hospital stay and could not be resuscitated [11]. Aljehani *et al*. reported a case of *C. albidus* fungaemia and meningitis in September 2023 in a preterm infant, linked to premature birth, prolonged antibiotics and invasive medical procedures. CSF was negative for India ink and the cryptococcal antigen test. Although HIV-negative, the infant’s condition was exacerbated by intravenous lines and total parenteral nutrition. Treatment with liposomal amphotericin B, excluding flucytosine, proved effective [12].

This case highlights the emerging role of *C. albidus* as a pathogen in immunocompromised patients, particularly those with T-ALL. The patient’s clinical improvement following antifungal therapy with liposomal amphotericin B and flucytosine underscores the importance of early diagnosis and appropriate treatment. Additionally, elevated procalcitonin levels, typically associated with bacterial infections, suggest a potential role for procalcitonin as a biomarker in fungal infections. This case emphasizes the need for heightened awareness of rare pathogens in meningitis diagnosis and management, particularly in immunosuppressed individuals, and calls for further research to explore the epidemiology, virulence and optimal treatment strategies for *C. albidus* infections.

## Abbreviations

AST: antimicrobial susceptibility testing
BHI: brain heart infusion
CLSI: Clinical and Laboratory Standards Institute
CNS: cerebrospinal fluid
CSF: cerebrospinal fluid
CT: computed tomography
HSV: herpes simplex virus
ME panel: Meningitis/Encephalitis panel
MRI: magnetic resonance imaging
SDA: Sabouraud dextrose agar
T-ALL: T-cell acute lymphoblastic leukaemia
